# Dropsy of the Amnion

**Published:** 1876

**Authors:** W. H. Byford

**Affiliations:** Professor of Obstetrics, Chicago Medical College


### DROPSY OF THE AMNION.

By W. H. BYFORD, A.M., M.D., Professor of Obstetrics, Chicago Medical College.

In most cases, dropsy of the amnion occurs only in the later months of pregnancy, and rarely in a degree sufficient to be more than a cause of tedious labor.

The tediousness in such cases is, doubtless, owing in part to the enfeebled condition of the uterine muscular fibres from over distention; more especially is it due to resistence of the tough membranes which render the accumulation possible.

While a moderate excess of liquor amnii at such a time is not uncommon, a great and dangerous dropsical distention of the amnion in the early part of gestation is very rare.

This latter condition undoubtedly is often prevented by the distention provoking powerful action of the uterus, and thus inducing abortion.

I do not find a case of early and extreme dropsy of this membranous sac reported in the transactions of the London Obstetric Society from the time of the organization of that institution to the present. Dr. Hodge, in his great work on Obstetrics, ignores it; while Cazeaux and Leishman only compile a short account of it, and leave the impression that neither of them has seen it.

I desire to call attention to a case that recently came under my observation, in which the accumulation was so great as to endanger the life of the patient and call for instant and decided means of relief.

The patient was an Irish woman, thirty-three years of age, the mother of three children, the youngest of which was two years old. She had always enjoyed good health, and passed through her previous pregnancies and labors without the occurrence of any unusual circumstances. When I saw her, August 30th, 1872, she supposed herself to be five months pregnant, and had for several weeks perceived fœtal movements.

About two months previously she had noticed enlargement of the abdomen, and since that time had increased in size very rapidly. When I first saw her, she presented the appearance of a patient with a very large multilocular ovarian tumor. The distension was so great that it caused much difficulty in breathing, and materially embarrassed the circulation.

The tumor extended up to and under the ribs, crowding the diaphargm high up into the thorax, displacing the heart upward, and pressing the costal cartilages outward nearly to a horizontal elevation.

The tumor was not definitely globular in its outline, but filled every portion of the abdomen and appeared to be packed into all the irregularities of that cavity. Its anterior portion was very promnient and uneven in contour, with several globular elevations of different sizes. One of these, and perhaps the largest, occupied the epigastric and a part of the right hypochondriac regions, the appearance of which strongly resembled one of the cystic prominences so often observed in the multilocular ovarian tumor.

To the left and a little below the umbilicus, a similar but smaller eminece could be seen, while the whole umbilical region was occupied by what seemed to be another very large cystic projection. The lumbar regions were prominent but dissimilar in contour.

The varied postures which the patient was directed to assume made no difference in the shape of the tumor.

Percussion elicited no resonance in any part of the abdomen, except very near the spine on both sides.

Fluctuation was apparent in every direction, but not equally. It was very indistinct anteriorly below the umbilicus, when percussion was made on the side of the tumor, but was quite quite distinct when the two hands were near together in this neighborhood.

From opposite sides, on a level with the umbilicus, very decided fluctuation could be elicited. Fluctuations could also be perceived distinctly in any distant part of the tumor when percussion was made over the epigastric elevation, or on the hypogastric region. No fluctuation was felt when the hand was placed over the resonant lumbar regions.

Upon making a vaginal examination, the uterus was found to be very low in the pelvis, the mouth far back toward the sacrum and sufficiently open to admit the finger, and the membranes were very tense. I could feel the head of a fœtus anterior to the cervix, and perform ballottment very easy; thus demonstrating the existence of pregnancy.

By passing two fingers high up in the left side of the pelvis, manifest fluctuation could be detected by them when percussion was made upon the upper part of the tumor.

The general aspect of the patient indicated great distress; her breathing was hurried and imperfect, and her countenance was suffused; the pulse was accelerated and feeble; the feet and legs were œdematous; the skin dry but not unnaturally warm, and the patient complained of great thirst. For two nights she had been unable to assume the horizontal position from a sense of impending suffocation, and had slept but little in the sitting posture.

Drs. J. N. Lilly and E. O. F. Roler met me in consultation at 2 P. M., August 31st.

There was no difficulty in ascertaining that all the symptoms were caused by a great accumulation of fluid in the abdomen, but what was the containing cavity was not so easily determined. The appearances alone, indicated, as we thought, the complication of a multilocular ovarian tumor with pregnancy. We did not think of ascites, and dropsy of the amnion was not certain. By evacuating the fluid we could most likely arrive at a correct diagnosis.

The idea of paracentesis, although discussed, was not entertained for fear of puncturing the impregnated uterus; and we determined to evacuate that organ for the purpose of making one step in the diagnosis, if it did not relieve the patient.

With the female catheter I ruptured the membranes and removed a pint or more of fluid, and through the rupture could feel the naked feet of a fœtus.

As an elastic and very tense substance was still to be felt in the os uteri, and I was not certain of its nature, I requested Dr. Lilly to make an examination. He declared he could feel the unruptured membranes, and that I was mistaken in supposing I had penetrated the bag of waters. I requested him to finish the work. After making strenuous efforts to do so with his fingers without success, I gave him the catheter, and it required considerable force to pass the instrument through the membranes. When he succeeded the bed was instantly deluged with water, and the abdominal tumor diminished with great rapidity.

Soon the outline of the contracting uterus could be seen and felt through the abdominal walls. The evacuation and contraction continued, until the uterus assumed its ordinary shape and size at five months' pregnancy. Thus the diagnosis and relief to the patient were both accomplished.

Squibb's fluid extract of ergot, in doses of thirty minims every two hours, was prescribed, and she was left in the care of Dr. Lilly, whose patient she was. After a short rest the uterus began to contract vigorously, and in about two hours two fœtuses, with their placentæ and membranes, were expelled. Dr. Hutchinson, who was called to the patient on account of an unavoidable absence of Dr. Lilly, made a careful examination of the expelled contents of the uterus, and assured me that, with the exception of the unequal development of the two membranous sacs, he could find nothing unusual about them. The fœtuses were equal in size and very like each other in appearance. Taking everything into consideration, it was evident that the membranous cavity of one fœtus was normal, while that of the other was distended with an enormous accumulation of amniotic fluid, and the membranes themselves were abnormally dense and tough. Much of the difficulty in arriving at a correct diagnosis in this case was caused by the great irregularity in shape and extreme amount of the distension. The early occurrence and rapidity of increase of the accumulated fluid, also produced uncertainty.

Cazeaux, who is closely followed by Lishman, says that dropsy of the amnion rarely occurs before the fifth month of pregnancy. In this case it commenced about the end of the third month, and attained a dangerous magnitude in the space of seven or eight weeks.

The two authors just mentioned speak of the difficulty of distinguishing between ascites and dropsy of the amnion, but do not consider the likelihood of confounding the latter affection with ovarian dropsy. They give the general symptoms present in our patient in a marked degree, viz.: scanty urine, œdema of the lower extremities and genital organs, thirst and fever, as indicating the presence of ascites instead of dropsy of the amnion.

Dr. Washington L. Atlee, in his work of unsurpassed value on the general and differential diagnosis of ovarian tumors, speaking of dropsy of the amnion, says: "In this form of dropsy, therefore, it must be plain how closely it resembles encysted or ovarian dropsy in its physical characteristics In being rapid in its development also, it is not unlike acute cases of ovarian tumors. It differs, however, from that, in uniformly suspending menstruation, and by altering the shape of the cervix uteri in proportion to its development, rapidly expanding and obliterating it, which is readily ascertained by examination." He might also have added ballottment as a valuable differential sign.

Dr. Atlee details three cases, two of which he saw, and for the other he copied the notes of Dr. James W. Kerr, of York, Pennsylvania. One of the patients was five months advanced in pregnancy with twins, when spontaneous miscarriage took place. He estimated the amount of amniotic fluid discharged at three gallons. His other patient was seven months pregnant with quadruplets, when the four sets of membranes ruptured successfully and spontaneously, and the whole contents of the uterus were expelled. The case communicated to him was mistaken for peritoneal dropsy, and tapped at the end of six months. Thirty-two pints of amniotic fluid were evacuated, when the uterus contracted down to its usual size at the six month of pregnancy. In a few days the fluid was accumulating so rapidly that it became a question whether the operation should not be repeated, as all the urgent symptoms were returning. Two weeks, however, from the time the fluid had been evacuated by the tapping, spontaneous expulsion ended the difficulty. The woman recovered rapidly from her confinement, and during the year and a half which had elapsed, at the time of writing, had enjoyed excellent health.

Dropsy of the amnion is not mentioned as one of the fluid accumulations in the abdomen, liable to be mistaken for ovarian tumor, by Mr. Spencer Wells, in his work on ovarian tumors, or Dr. Peaslee, in his book on the same subject.—*Chicago Medical Journal and Examiner.*

